# Guillain-Barré syndrome presenting with abdominal pain as the initial manifestation: a case report

**DOI:** 10.3389/fpain.2026.1839656

**Published:** 2026-06-11

**Authors:** Zi-Zhen Yin, Yu Liu

**Affiliations:** 1 Department of Hepatobiliary Surgery, Heze Medical College, Heze, Shandong, China; 2 Department of Internal Medicine, Central Hospital in Mudan District of Heze, Heze, Shandong, China

**Keywords:** abdominal pain, albuminocytological dissociation, Brighton criteria, case report, Guillain Barré syndrome, intravenous immunoglobulin, plasma exchange

## Abstract

**Background:**

Guillain-Barré syndrome (GBS) is an acute immune-mediated polyneuropathy, characteristically presenting with ascending limb weakness and sensory disturbances. However, GBS presenting with abdominal pain as the initial manifestation is relatively rare, abdominal pain is not a characteristic manifestation, its occurrence may delay diagnosis of GBS.

**Case presentation:**

A 62-year-old male patient presented to our hospital in January 2024 with a 5-day history of abdominal pain in the right upper quadrant. He was initially diagnosed with acute cholecystitis. However, the patient subsequently developed progressive limb weakness and loss of tendon reflexes, along with worsening respiratory distress and chest tightness, ultimately leading to a diagnosis of GBS. This case report describes a severe case of GBS presenting with acute abdominal pain as the initial manifestation, highlighting the diagnostic challenges of GBS in the setting of acute abdominal pain.

**Conclusion:**

Abdominal pain may precede characteristic neurological symptoms in GBS. GBS presenting with acute abdominal pain as the initial manifestation may pose diagnostic challenges and lead to delayed diagnosis. Physicians should maintain a high index of suspicion for GBS in patients with abdominal pain and rapid neurological deterioration.

## 1 Introduction

Acute abdominal pain is a condition commonly managed in clinical practice, whereas GBS is an immune-mediated acute inflammatory peripheral neuropathy. Acute abdominal pain as the initial manifestation of GBS is uncommon, posing significant diagnostic challenges for physicians and potentially leading to delayed diagnosis. In January 2024, a male patient initially presenting with acute abdominal pain in the right upper quadrant was initially diagnosed with acute cholecystitis. However, the patient subsequently developed progressive limb weakness and absent tendon reflexes, accompanied by worsening respiratory distress and chest tightness, ultimately leading to a diagnosis of GBS. He was subsequently transferred to the neurological intensive care unit. Physicians may be unfamiliar with GBS presenting with acute abdominal pain as its initial manifestation. Therefore, we present this case to enrich the clinical literature and assist colleagues in identifying such rare conditions.

## 2 Case presentation

A 62-year-old male patient presented to our emergency department on the night of January 8, 2024, with acute abdominal pain in the right upper quadrant, accompanied by fever for 5 days, with the highest temperature reaching 38.0℃. Abdominal ultrasound revealed an enlarged gallbladder along with surrounding fluid accumulation, which led to his hospitalization.

**Figure 1:**
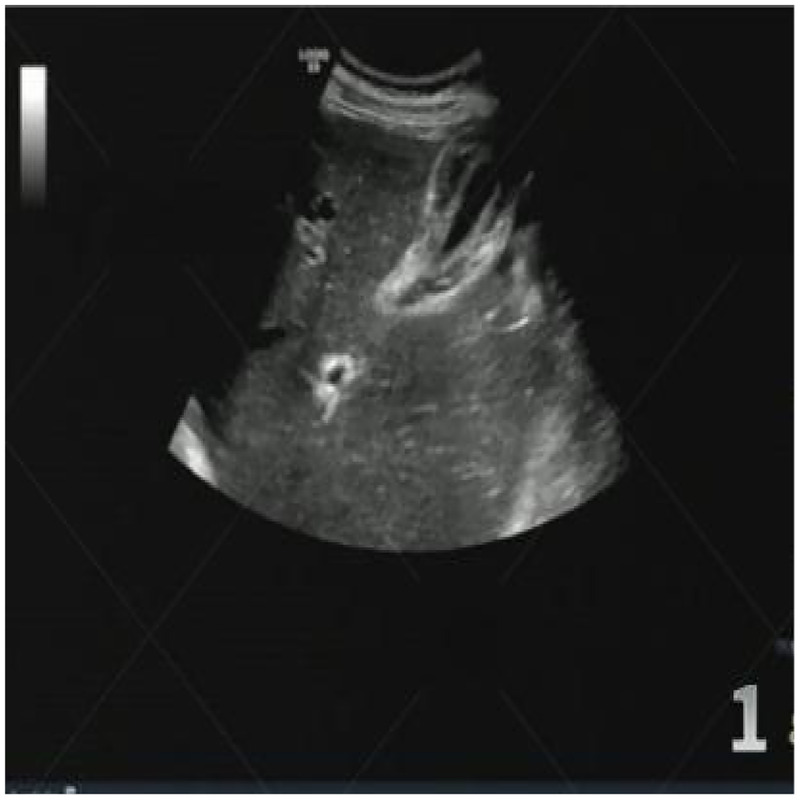
Ultrasound revealed edema of the gallbladder wall.

**Figure 2:**
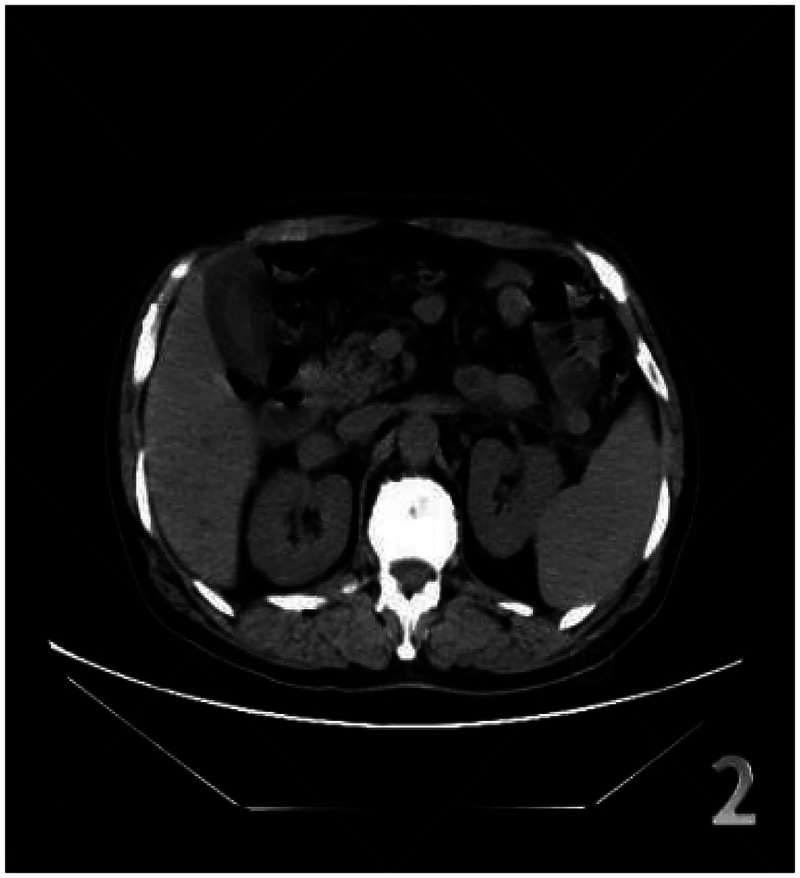
CT revealed increased gallbladder density.

**Figure 3:**
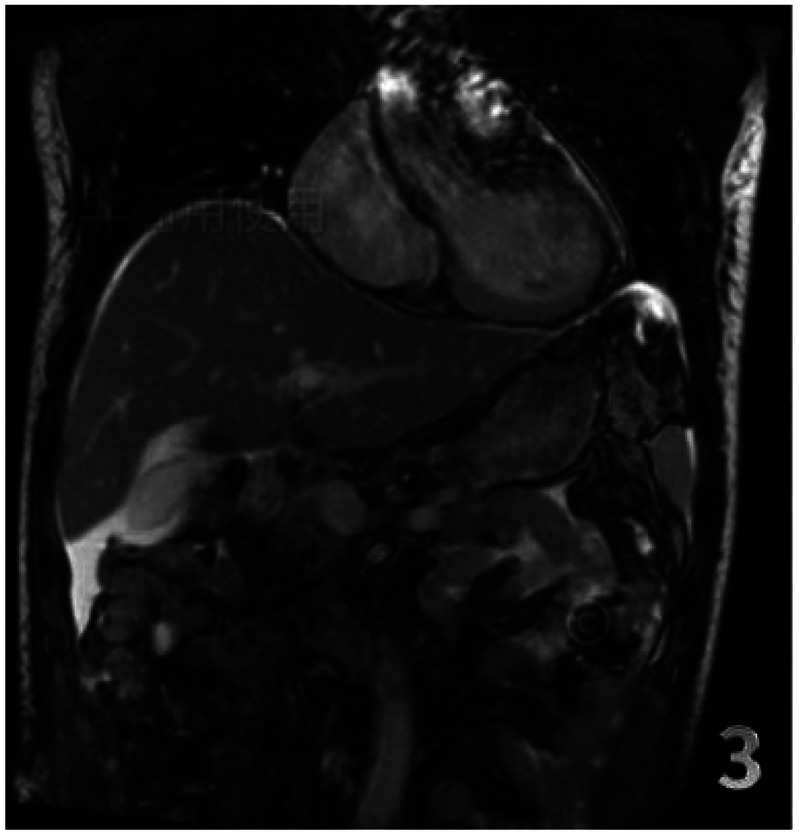
MRI showed pericholecystic fluid collection.

The patient reported experiencing a cold approximately 1 month ago. He was prescribed routine medication for his cold and discontinued treatment following resolution of clinical symptoms. He has no prior history of jaundice, cardiovascular disease, hypertension, or diabetes. He has not received any vaccine recently. Additionally, he occasionally smokes and consumes alcohol. The patient did not have any relevant family medical history. Tenderness was noted in the right upper quadrant of the abdomen, and Murphy's sign was positive. Bowel sounds were diminished. No obvious dry or moist rales were heard on lung auscultation. A comprehensive set of laboratory examinations was conducted, including a complete blood count; C-reactive protein; electrolytes; blood glucose levels; cardiac enzymes; cardiac troponin; myoglobin; and tests to evaluate thyroid, liver, kidney, and pancreas function. The white blood cell count was 12.13 × 10^9^/L, and the C-reactive protein level was 24.55 mg/L. Liver function tests revealed alanine aminotransferase at 119.1 U/L, aspartate aminotransferase 103.2 U/L, *γ*-glutamyl transferase 107.4 U/L, alkaline phosphatase 138.5 U/L, total bilirubin 36.6 umol/L, blood glucose 8.1 mmol/L, all above their respective upper limits of normal levels. His potassium was 4.1 mmol/L, and sodium 140.0 mmol/L. Other results related to thyroid function, kidney function, pancreatic function, electrolytes, cardiac enzymes, myoglobin, and cardiac troponin were within normal ranges. Electrocardiographic findings demonstrated ST-segment changes, prolonged QT interval, and abnormal Q waves. Ultrasound revealed significant edema and thickening of the gallbladder wall (Figure 1). Computed tomography (CT) demonstrated gallbladder wall thickening alongside increased intraluminal density (Figure 2), while magnetic resonance imaging (MRI) findings confirmed a pericholecystic fluid collection (Figure 3).

Ultrasound, CT, and MRI images revealed no evidence of cholelithiasis, and no imaging features suggestive of portal hypertension or liver cirrhosis were identified. Based on these findings, the patient was diagnosed with acute acalculous cholecystitis; He was instructed to refrain from oral intake and received intravenous fluids along with empiric broad-spectrum antibiotics. Laparoscopic cholecystectomy was scheduled.

The following morning, the patient's abdominal pain was rapidly alleviated after intravenous therapy. The patient's anti-inflammatory treatment was highly effective. But the patient reported limb weakness. We found his lower limbs demonstrated the ability to lift off the bed surface and resist mild resistance, while his upper limbs exhibited preserved mobility with a strong hand grip. At that time, we hypothesized that fasting might have contributed to the observed weakness. In the afternoon, hyporeflexia was noted on physical examination. Brain and spine MRI was performed to rule out cerebral infarction; however, no ischemic lesions were identified. Unfortunately, this progressive neurological deterioration did not receive adequate clinical attention at that time.

On day 2, the patient reported limb weakness and chest oppression. Antiasthmatic medication was administered; however, the therapeutic response was unsatisfactory. The patient subsequently developed paresthesia in the hands and feet, progressive limb weakness resulting in an inability to ambulate, and rapid deterioration in general condition. Further neurological examination revealed absent tendon reflexes, dysphagia, and episodes of choking while drinking water. Muscle strength in the lower limbs declined to 1/5, and that in the upper limbs decreased to 3/5. Given the atypical presentation, the clinical team sought consultation with neurologists. Following a thorough physical examination and clinical assessment, acute myelitis was ruled out due to the absence of bladder or bowel dysfunction, and a definitive diagnosis of GBS was established.

Due to progressive respiratory distress accompanied by chest tightness, laparoscopic cholecystectomy and gallbladder puncture drainage were contraindicated. The patient was subsequently transferred to the neurological intensive care unit. He subsequently received mechanical ventilation, plasma exchange, intravenous immunoglobulin at adequate dosing, and high-dose methylprednisolone therapy. Electrophysiological studies demonstrated reduced motor and sensory nerve conduction velocities, consistent with peripheral nerve demyelination and axonal involvement. A sural nerve biopsy revealed demyelination and inflammatory cell infiltration, findings that served as a key diagnostic reference supporting the diagnosis of GBS. Cerebrospinal fluid (CSF) analysis was performed. Initially, CSF protein levels were within the normal range. However, 3 weeks later, the symptoms were effectively managed and demonstrated gradual improvement. At this stage, CSF protein level was elevated to 1.85 g/L, while the white blood cell count remained normal. This finding is consistent with albuminocytological dissociation, a hallmark of GBS.

Four weeks after treatment initiation, the patient achieved independent respiration without mechanical ventilation support and was able to consume a full bowl of millet porridge per meal without difficulty. He also exhibited partial recovery of sensory function in the fingers and toes, with initial signs of improved limb strength, although he remained bedridden at this stage. Gradual rehabilitation training was initiated, and the patient was ultimately discharged from the hospital following 2 months of comprehensive treatment.

Follow-up assessments were conducted at 6, 9, and 12 months post-discharge. At the 6-month evaluation, the patient demonstrated marked improvement in motor function, with the ability to walk independently using a cane for distances up to 500 meters and to perform basic activities of daily living, such as feeding, without assistance. Sensory deficits in the extremities were nearly resolved, with only occasional mild paresthesia reported during cold weather. The patient no longer experienced pain in the right upper quadrant, and ultrasound examination did not reveal pericholecystic fluid. By the 9-month assessment, the patient had attained independent ambulation, having discontinued cane use and demonstrating the capacity to walk steadily for distances up to 1 kilometer without fatigue. Residual numbness and mild muscular atrophy persisted, but neurological examinations revealed normal reflexes and full range of motion in all limbs. The patient no longer experiences discomfort in the right upper quadrant. At the 12-month follow-up, the patient exhibited significant gains in functional independence. and muscular function was nearly fully restored. The patient had completely regained pre-illness levels of motor and sensory function. No adverse events or recurrence of the initial condition were observed during any follow-up visit, and all laboratory tests and imaging studies remained within normal limits.

## 3 Discussion

Acute abdominal pain is a condition commonly managed in clinical practice; whereas GBS is an immune-mediated acute inflammatory peripheral neuropathy that often manifests as a life-threatening disease (1). Acute abdominal pain as the initial presentation of GBS is rare. In January 2024, we managed a GBS patient presenting with acute abdominal pain as the initial manifestation. Unfortunately, he was diagnosed with acute cholecystitis due to abdominal pain in the right upper quadrant, resulting in delayed recognition and suboptimal treatment initiation. Herein, we present this case for further study and discussion.

The incidence of acute cholecystitis is gradually increasing among older individuals (2), its characteristic symptom is abdominal pain in the right upper quadrant. Our patient presented with right upper quadrant abdominal pain, combined with imaging studies and laboratory results, he was diagnosed with acute acalculous cholecystitis based on the diagnostic criteria for acute cholecystitis (3). However, the patient subsequently developed progressive limb weakness and loss of tendon reflexes, along with worsening respiratory distress and chest tightness. His cerebrospinal fluid analysis revealed protein-cell dissociation. Electrophysiological studies demonstrated reduced motor and sensory nerve conduction velocities, and sural nerve biopsy confirmed demyelination with inflammatory cell infiltration. The patient fulfilled the Brighton criteria for GBS, level 1 diagnostic certainty was fulfilled (4) (Table 1). The patient was finally diagnosed with GBS.

**Table 1:** The Brighton criteria of Guillain-Barré syndrome.

Diagnostic criteria	1	2	3	4
Bilateral flaccid weakness of the limbs	+	+	+	+/−
Decreased or absent deep tendon reflexes in the affected limbs	+	+	+	+/−
Monophasic course and onset period of 12 h to 28 d	+	+	+	+/−
Cell count in CSF of <50/uL	+	+/−a	−	+/−
CSF protein concentration greater than normal value	+	+/−a	−	+/−
NCS findings consistent with one of the subtypes of GBS	+	+/−	−	+/−
Absence of an alternative diagnosis for weakness	+	+	+	+

+, present; −, absent; +/−, present or absent; CSF, cerebrospinal fluid; GBS, Guillain-Barré syndrome; NCS, nerve conduction study.

If CSF is not collected or data can not be obtained, neurophysiological data should be consistent with the diagnosis of GBS.

GBS is an acute and demyelinating polyradiculoneuropathy, clinically characterized by progressive and symmetric limb weakness accompanied by absent or diminished deep tendon reflexes (5, 6). The Brighton criteria facilitate the early and accurate diagnosis and play a crucial role in the detection of GBS (7). Conditions such as hypokalemia and myasthenia gravis are distinct clinical entities that can be differentiated from GBS through careful clinical assessment. Acute transverse myelitis represents a critical differential diagnosis. It typically manifests with sudden onset of flaccid paraplegia, reduced muscle tone, absent tendon reflexes, and a clearly defined sensory level, often accompanied by positive pathological reflexes and bladder or bowel dysfunction. In contrast to GBS, involvement of the upper limbs is uncommon in transverse myelitis. CSF analysis in transverse myelitis may reveal mild pleocytosis, whereas protein levels generally remain within the normal range. Combined with the cerebrospinal fluid examination and spine MRI imaging features, acute transverse myelitis and other neurological diseases were excluded.

The diagnosis of GBS is typically established based on clinical patterns (8). The annual incidence of GBS is one or two cases per 100000 individuals (9). CSF analysis can provide valuable support, albuminocytological dissociation is a hallmark feature of GBS (10, 11). Intravenous immunoglobulin is often considered the first-line therapy for practical reasons (12, 13), both plasma exchange and high-dose intravenous immunoglobulin have demonstrated efficacy in reducing disease severity and residual neurological deficits (14, 15). Approximately 80%–85% of cases achieve a full functional recovery (16). Nevertheless, GBS is a life-threatening disease, about one-third of patients develop respiratory insufficiency, requiring prolonged intensive care or mechanical ventilation (17), mortality rates are estimated at 5% (18).

Regrettably, in this instance, acute abdominal pain in the right upper quadrant led to a diagnosis of acute cholecystitis, GBS was not established promptly. Delayed diagnosis of GBS stemmed from three interrelated factors: insufficient clinical recognition of its potential co-occurrence with acute abdominal pain; the absence of established diagnostic criteria for this rare neurological complication; and the masking of early neurological signs by prominent acute abdominal symptoms.

GBS primarily affects the peripheral nervous system and may also involve autonomic nerves. Autonomic dysfunction has been reported in more than half of GBS cases (19–21), potentially leading to dysfunction of multiple internal organs, particularly affecting the cardiovascular, gastrointestinal, and urinary systems. Patients may present with a range of clinical manifestations, including arrhythmias, gastrointestinal dysmotility, and other autonomic disturbances. Some researchers reported paralytic ileus as the initial presentation of GBS (22, 23). In our patient, gastrointestinal dysfunction was absent; instead, the presentation manifested as abdominal pain in the right upper quadrant, setting it apart from previously reported cases. Abdominal pain may occur before limb weakness. There have been a few documented cases in which abdominal pain was the initial presenting symptom (24–27). Abdominal pain in GBS may be triggered by sensory nerve inflammation, dorsal nerve root inflammation, and gastrointestinal autonomic dysfunction (27). Because abdominal pain is not a characteristic manifestation of GBS, cases initially presenting with acute abdominal pain are rare, leading to this patient's diagnosis of acute cholecystitis. Acute abdominal pain or acute cholecystitis represented as a secondary or associated condition rather than a truly independent disease. The occurrence of abdominal pain delayed the diagnosis of GBS and posed a significant diagnostic challenge for physicians.

It has long been established that GBS is a post-infection autoimmune disorder affecting the peripheral nervous system. However, the immunological mechanisms underlying GBS pathogenesis remain elusive in a significant proportion of patients. Antecedent infections and the phenomenon of molecular mimicry, in which antibodies generated against infectious agents exhibit cross-reactivity with host gangliosides, are widely acknowledged as the primary mechanisms contributing to GBS (28). Gangliosides are critical antigenic targets in GBS pathogenesis (29). Previous studies have identified anti-ganglioside antibodies (e.g., anti-GM1, anti-GD1b) in GBS patients presenting with severe gastrointestinal autonomic dysfunction, including delayed gastric emptying and intestinal pseudo-obstruction (30, 31). These findings suggest that cross-reactive antibodies in GBS may potentially target gangliosides on gallbladder innervating nerves, disrupting synaptic transmission and impairing smooth muscle contraction. Current medical evidence indicates that the immune attack in GBS has systemic features. We speculate that GBS may affect the neuromuscular function of the gallbladder, potentially leading to a weakened gallbladder contractility. This dysfunction may subsequently result in gallbladder dilation and clinical manifestations consistent with biliary stasis and cholecystitis.

GBS manifesting with acute abdominal pain as the initial symptom is exceedingly uncommon, imposing considerable diagnostic hurdles for physicians and heightening the risk of delayed recognition and management. The abdominal pain the patient reported was atypical for GBS, it diverted physicians' attention and heightened the diagnostic difficulty. The pathophysiological mechanism underlying acute abdominal pain as the initial presentation of GBS remains unclear. It took us 2 days from the patient's admission to the final diagnosis of GBS. Early recognition and prompt initiation of treatment are crucial in improving outcomes and reducing mortality in GBS (32). We present this case to contribute to the existing body of knowledge and to support enhanced diagnostic precision and clinical decision-making in future practice.

## 4 Conclusion

GBS presenting with acute abdominal pain as the initial manifestation can present significant diagnostic challenges. This acute abdominal presentation may mask early neurological signs of GBS, resulting in delayed diagnosis and treatment initiation.

## Data Availability

The original contributions presented in the study are included in the article/Supplementary Material, further inquiries can be directed to the corresponding author.
